# Bioelectrochemical production of hydrogen in an innovative pressure-retarded osmosis/microbial electrolysis cell system: experiments and modeling

**DOI:** 10.1186/s13068-015-0305-0

**Published:** 2015-08-14

**Authors:** Heyang Yuan, Yaobin Lu, Ibrahim M Abu-Reesh, Zhen He

**Affiliations:** Department of Civil and Environmental Engineering, Virginia Polytechnic Institute and State University, Blacksburg, VA 24061 USA; Department of Chemical Engineering, College of Engineering, Qatar University, P.O. Box 2713, Doha, Qatar

## Abstract

**Background:**

While microbial electrolysis cells (MECs) can simultaneously produce bioelectrochemical hydrogen and treat wastewater, they consume considerable energy to overcome the unfavorable thermodynamics, which is not sustainable and economically feasible in practical applications. This study presents a proof-of-concept system in which hydrogen can be produced in an MEC powered by theoretically predicated energy from pressure-retarded osmosis (PRO). The system consists of a PRO unit that extracts high-quality water and generates electricity from water osmosis, and an MEC for organic removal and hydrogen production. The feasibility of the system was demonstrated using simulated PRO performance (in terms of energy production and effluent quality) and experimental MEC results (e.g., hydrogen production and organic removal).

**Results:**

The PRO and MEC models were proven to be valid. The model predicted that the PRO unit could produce 485 mL of clean water and 579 J of energy with 600 mL of draw solution (0.8 M of NaCl). The amount of the predicated energy was applied to the MEC by a power supply, which drove the MEC to remove 93.7 % of the organic compounds and produce 32.8 mL of H_2_ experimentally. Increasing the PRO influent volume and draw concentration could produce more energy for the MEC operation, and correspondingly increase the MEC hydraulic retention time (HRT) and total hydrogen production. The models predicted that at an external voltage of 0.9 V, the MEC energy consumption reached the maximum PRO energy production. With a higher external voltage, the MEC energy consumption would exceed the PRO energy production, leading to negative effects on both organic removal and hydrogen production.

**Conclusions:**

The PRO-MEC system holds great promise in addressing water-energy nexus through organic removal, hydrogen production, and water recovery: (1) the PRO unit can reduce the volume of wastewater and extract clean water; (2) the PRO effluents can be further treated by the MEC; and (3) the osmotic energy harvested from the PRO unit can be applied to the MEC for sustainable bioelectrochemical hydrogen production.

**Electronic supplementary material:**

The online version of this article (doi:10.1186/s13068-015-0305-0) contains supplementary material, which is available to authorized users.

## Background

Microbial electrolysis cells (MECs) is an attractive technology that can simultaneously remove organics and produce hydrogen gas. In MECs, exoelectrogens growing on the anode respire by releasing electrons extracellularly; driven by an external voltage >0.2 V, those electrons flow to the cathode to reduce protons into hydrogen gas [1]. MECs are of strong interests because its energy consumption could be significantly lower than that of conventional methods such as water-splitting and steam reforming [2, 3]. Life cycle assessment suggested that MECs might outperform the prevailing wastewater treatment methods (i.e. activated sludge process and anaerobic digestion) in terms of energy requirement, greenhouse gas effect and other environmental impacts [4, 5]. However, the requirement of additional energy, which is mostly from fossil fuels, should be further addressed to make MECs more sustainable.

Researchers have explored alternative energy sources to drive hydrogen production in MECs. For example, a microbial fuel cell (MFC) was used to replace external power supply and provided a voltage to achieve a hydrogen production rate of 0.015 m^3^ m^−3^ d^−1^ in an MFC-MEC coupled system [6]. It was found that the hydrogen yield was relatively low and also instable in this coupled system, possibly due to cell voltage reversal between the two bioelectrochemical systems (BES). To avoid voltage reversal, a capacitor circuit was installed between MFCs and an MEC, and helped achieve 38 % higher hydrogen production rate compared to the directly coupled system [7]. In another study, solar energy was harvested by a dye-sensitized solar cell and then applied on an MEC, which achieved a hydrogen production rate of 0.07 m^3^ m^−3^ d^−1^ [8]. The entropic energy stored in a salinity gradient between seawater and fresh water is estimated to be 0.8 kW m^−3^ [9, 10], which could be captured by reverse electrodialysis and then used to drive MECs [11–13].

Unlike salinity energy that relies on salt movement, osmotic energy can be produced through water interaction between saline water and freshwater, and can be harvested by using pressure-retarded osmosis (PRO). In a PRO system, water is driven by the salinity gradient and diffuses from a low-salinity solution (feed) to a high-salinity solution (draw) through a semi-permeable membrane; consequently, electrical energy is generated by pressurizing the diluted draw (whose volume becomes larger after water extraction) through a hydroturbine [14]. As a forward osmosis (FO) process, PRO shows low fouling propensity compared to reverse osmosis because of relatively low water flux and hydraulic pressure [15]. The recent advances in membrane technology could greatly reduce the capital cost of PRO and thus make it competitive with other renewable energy technologies [16]. The highest power density produced by PRO was reported to be 10.6 W m^−2^ with 1 M NaCl solution as draw [17].

We have previously integrated osmotic processes with BES to assist with bioelectricity generation in MFCs or desalination in microbial desalination cells (MDCs) [18]. For example, the first osmotic microbial fuel cell (OsMFC) was developed by replacing the ion exchange membrane with a FO membrane, and accomplished simultaneous wastewater treatment, water extraction, and bioenergy production [19]. The performance of an OsMFC was affected by its draw solution, membrane condition, anode substrates, and cathode reactions [20–22]. The osmotic process was integrated with MDCs in several ways: replacement of anion exchange membrane with an FO membrane to create an osmotic MDC, linking an OsMFC with an MDC, or connecting an FO to an MDC [23–25]. Those prior studies have demonstrated the synergy between BES and FO, and provided a foundation for the present study.

**Fig. 1:**
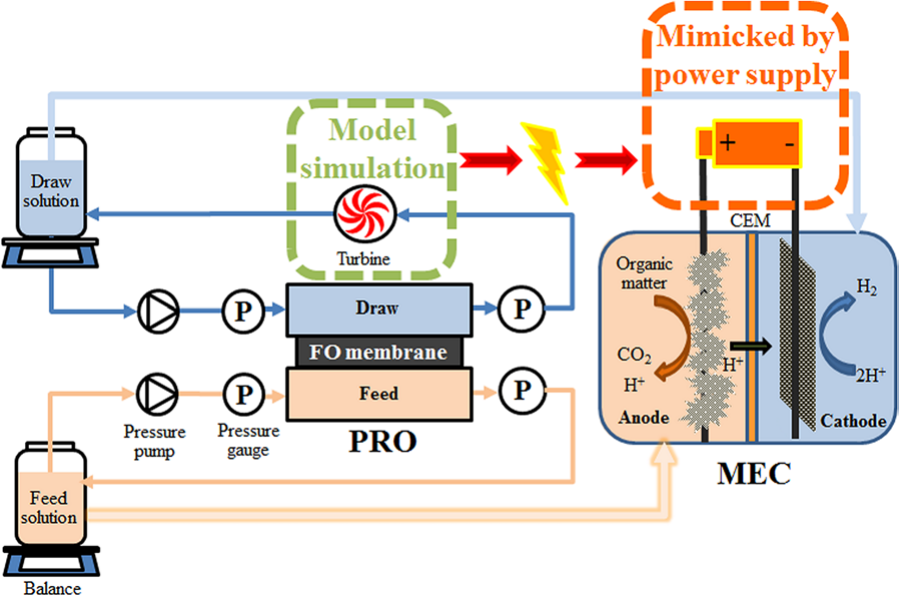
Schematic of the proposed system consisting of a PRO unit and an MEC through hydraulic connection.

Given the fact that PRO can generate electric energy and MECs need additional energy for hydrogen production, a PRO-MEC system is proposed here: wastewater (feed) and seawater (draw) flow into a PRO unit for water extraction and energy generation; then, the energy is applied to an MEC for organic removal and hydrogen production, with the PRO feed and draw effluents serving as the anolyte and the catholyte of the MEC, respectively (Fig. 1). This is the first time that the PRO energy (osmotic-to-electricity) is studied to drive the conversion of organic compounds to hydrogen in an MEC; in addition to electrical interaction between the two components, this system also contains hydraulic linkage between the two for wastewater treatment and reuse and saline water desalination. After further desalination, the produced water (diluted draw solution) could be useful for non-potable applications such as agricultural irrigation and landscaping. The system may have application niches in a location with seawater and a high demand for wastewater reuse.

To examine the proposed system, we first developed a time-dependent PRO model and a batch-mode MEC model. According to the simulated water flux obtained from the PRO model, the anolyte and the catholyte were prepared for an MEC that was operated experimentally using a power supply to mimic the energy supply process. The system feasibility was demonstrated by comparing the simulated PRO energy production and the experimental MEC energy consumption, together with other parameters such as water extraction, organics removal and hydrogen production. Finally, the PRO and MEC models were used to predict the system performance at varied influent volume, draw concentration and external voltage.

## Results and discussion

### Model validation

Mathematical modeling can help understand the key factors in the PRO-MEC system and predict the behaviors that cannot be easily investigated by experiments. Previous PRO studies focused on achieving instantaneous maximum energy production, and it was not necessary to take the concentration change of the feed and the draw into account because of the constant replenishment of fresh solutions to maintain high osmotic pressure difference [26, 27]. However, that is not applicable to a PRO-MEC system, because the relatively fast PRO process will generate excessive amount of effluent that can exceed the treating capacity of bench-scale MECs. In this regard, the PRO unit must be operated under a batch mode and the time variable must be introduced in the PRO model to predict the water flux and energy production. To examine that hypothesis, the PRO unit was operated for 6.0 h at different hydraulic pressures (1, 2 and 3 bar) with the initial conditions listed in Table 1. At 1 bar, the feed solution decreased from 600 mL to 138 mL (462 mL of water recovery) with significant decrease in water flux (Additional file 1). The experiments at 2 and 3 bar showed similar trends, but with slightly lower water recovery due to the higher hydraulic pressure (Additional file 1). With the PRO parameters listed in the Additional file 2, the time-dependent PRO model successfully predicted the volume profile with RMSE less than 2.5 % (Table 2). The experiments with different NaCl concentrations (0.5 and 2.0 M) at 1 bar were conducted to further validate the model. After 5.0-h operation, the feed volume decreased from 600 mL to 452 mL with 0.5 M (148 mL water recovery, Additional file 3), 65 % lower than that with 0.8 M at the same operation time because of the lower osmotic pressure difference between the feed and the draw. On the other hand, water recovery rapidly reached 315 mL in 2.0 h with 2.0-M NaCl solution (Additional file 3). The results collectively demonstrated that the time-dependent PRO simulation agreed well with the experimental data (Table 2).

**Table 1:** The initial conditions for the PRO experiments and simulation in different studies

Experiment	V_PRO_^a^ (mL)	S_PRO_^b^ (mg L^−1^)	NaCl_In_^c^ (M)	P^d^ (bar)	V_A_^e^ (mL)	V_C_^f^ (mL)	S_A_^g^	Voltage^h^ (V)
							(mg L^−1^)	
Exp1-model validation	600	193	0.5, 0.8 and 2.0	1, 2 and 3	115	1,085	357, 1,006 and 2,007	0.6, 0.8 and 1
Exp2-system feasibility	600	193	0.8	P_t_	115	1,085	1,007	0.8
Exp3-PRO influent volume	100–2,000	193	0.8	P_t_	Obtained from the time-dependent PRO model			0.8
Exp4-NaCl concentration	600	193	0.1–2.0	P_t_				0.8
Exp5-external voltage	600	193	0.8	P_t_	115	1,085	1,007	0.5–1.1

^a^Volume of PRO feed and draw influent.

^b^Substrate concentration in the PRO feed influent.

^c^NaCl concentration in the PRO draw influent.

^d^Hydraulic pressure applied on the PRO draw chamber.

^e^Volume of MEC anolyte influent.

^f^Volume of MEC catholyte influent.

^g^Substrate concentration in the MEC anolyte influent.

^h^External voltage applied on the MEC.

**Table 2:** RMSE for the PRO and MEC simulation

	RMSE (%)
PRO	
1 bar-feed	1.8 ± 0.3
1 bar draw	0.8 ± 0.0
2 bar-feed	1.9 ± 0.6
2 bar draw	0.8 ± 0.0
3 bar-feed	0.9 ± 0.1
3 bar draw	2.4 ± 0.6
0.5 M feed	1.9 ± 0.2
0.5 M draw	0.9 ± 0.0
2.0 M feed	1.4 ± 0.3
2.0 M draw	0.8 ± 0.0
MEC	
0.6 V	16.5 + 5.6
0.8 V	13.0 ± 2.3
1.0 V	23.6 + 1.3
357 mg L^−1^	12.1
2,007 mg L^−1^	21.6

The RMSE of the PRO model was calculated using the feed volume, and that of the MEC model was calculated using electricity.

Standard deviation was calculated with triplicate experiments.

Based on the method presented in previous studies of MDC and MEC modeling [28, 29], a batch-mode MEC model was implemented with independent experiments of varied substrate and external resistance. The introduction of [HCO_3_^−^] and [H^+^] into the anode potential (Eqs. 14, 15) and the substrate change in the concentration overpotential (Eq. 17) helped achieve satisfactory agreement between the experimental and simulation results. The RMSE for the MEC model (Table 2) was relatively high mainly due to the overestimation of organics removal (99.9 % removal) at the end of the MEC process. Consequently, at 0.8 V, the model yielded slightly higher energy consumption (488 J) and hydrogen production (36.7 mL) compared to the experiments (470 J and 32.8 mL). The MEC’s operation time was prolonged from 19.2 h at 357 mg L^−1^ acetate to 96.1 h at 2,007 mg L^−1^ acetate (Additional file 4). When the external voltage was increased, the current was improved from 2.7 mA at 0.6 V to 6.6 mA at 1.0 V (Additional file 4) with 60 % increase in Coulombic efficiency and 40 % increase in cathodic efficiency (Table 3). As a result, both hydrogen production and energy consumption increased by two times. The experimental results are consistent with previous studies [30]. In summary, the simulated current generation under different conditions could match the experimental data, with slight overestimation of organics removal, energy consumption and hydrogen production.

**Table 3:** The MEC performance at different external voltage

Voltage (V)	S_In_^a^ (mg L^−1^)	C_In_^b^ (mS cm^−1^)	V_H2_^c^ (mL)	Rate_H2_ (m^3^ m^−3^ d^−1^)	∆S^d^ (%)	R_CE_^e^ (%)	R_cat_^f^ (%)	Energy (J)	HRT (h)
0.6	1,009 ± 31	19.5 ± 0.8	18.4 ± 0.9	0.008 ± 0.001	95.6 ± 0.0	36.1 ± 0.0	42.9 ± 0.0	221 ± 7	54.4 ± 2.3
0.8	1,009 ± 8	18.3 ± 0.1	32.8 ± 0.6	0.016 ± 0.001	93.7 ± 0.0	57.5 ± 0.0	48.1 ± 0.0	470 ± 2	46.9 ± 1.4
1.0	987 ± 53	19.9 ± 1.5	40.8 ± 2.8	0.030 ± 0.005	94.7 ± 0.0	58.3 ± 0.0	60.7 ± 0.1	581 ± 22	30.9 ± 3.2

Standard deviation was calculated with triplicate experiments.

^a^Substrate concentration in the MEC influent.

^b^Conductivity of the MEC influent.

^c^Total H_2_ production.

^d^Organics removal.

^e^Coulombic efficiency.

^f^Cathodic efficiency.

### System feasibility

**Fig. 2:**
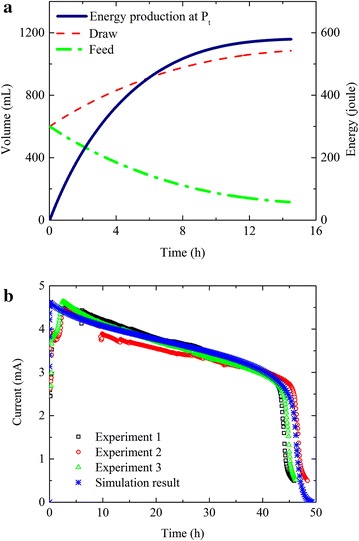
**a** Volume profile and energy production in the PRO unit at the hydraulic pressure *P*_*t*_ = (*π*_*D,t*_ − *π*_*F,t*_)/2; **b** experimental data and simulation result of current generation in the MEC at 0.8 V using electrolyte and energy produced by the PRO unit.

The feasibility of the PRO-MEC system was demonstrated by estimating energy production in the PRO unit using the models and applying the energy (mimicked by a power supply) to the MEC for hydrogen production and organic removal. It should be noted that, because of the difficulty in generating real energy from bench-scale PRO units at a high hydraulic pressure, the majority of the PRO studies or publications adopted theoretical estimate of energy production [31–34]. The results showed that the PRO unit could theoretically generate sufficient energy to drive the MEC, which successfully produced hydrogen gas and removed more than 90 % of organic compounds. In more details, the PRO simulation was performed at the hydraulic pressure of *P*_*t*_ = (*π*_*D,t*_ − *π*_*F,t*_)/2 (Eq. 7, at the maximum energy mode), and was stopped when water flux dropped below 0.5 LMH (L m^−2^ h^−1^). This was because that after 14.5 h of simulation, *P*_*t*_ decreased to 2.4 bar and both the volume and the energy production reached a plateau (Fig. 2a). At the end of the simulation, the volume of the feed solution decreased from the initial 600 mL to 115 mL and the draw volume increased to 1085 mL, suggesting that 485 mL of clean water was extracted from the feed solution. Meanwhile, the PRO unit could produce a feed effluent containing 1,007 mg L^−1^ acetate, a draw effluent with 0.46 M NaCl, and 579 J of energy (Table 1; Fig. 2a).

Based on those results obtained from the PRO simulation, 115 mL of anolyte with 1,009 ± 8 mg L^−1^ acetate and 1,085 mL of the catholyte with the 0.46 M NaCl were prepared and supplied to the MEC (Table 1). An external voltage of 0.8 V was applied to the MEC to mimic the utilization of the energy produced by the PRO unit. The MEC operating time was determined by equating the PRO energy production with the MEC energy consumption (Eqs. 8 and 19). The MEC current decreased steadily over time (Fig. 2b), mainly due to the consumption of organic compounds. At the end of the experiment, the MEC removed 93.7 % of the organics and produced 32.8 mL of H_2_ at the expense of 470 J of energy after 46.9 h (Table 3), indicating that the energy produced by the PRO unit (579 J) was sufficient to drive the MEC. The relatively low hydrogen production rate (0.016 m^3^ m^−3^ d^−1^) and cathodic efficiency (48.1 %) was probably because no buffer was added in the catholyte (the pH increased from neutral to 11.6 after 3.0 h of operation). Coulombic efficiency (57.5 %) was comparable with other MECs, likely benefited from a relatively high organics concentration and conductivity of the anolyte, which was the concentrated feed solution from the PRO unit. Similar to the substrate concentration, the conductivity in the feed was concentrated by 5.2 times due to water extraction in the PRO unit, resulting in an initial conductivity of 18.3 mS cm^−1^ in the MEC anolyte. All those results have demonstrated that it is possible to harvest osmotic energy in the PRO unit and use it to convert chemical energy into hydrogen gas in the MEC from the same organic solution. In addition, the coupling of PRO and MEC can simultaneously recover clean water and reduce the volume of the organic solution.

### Model prediction

#### Effects of PRO influent volume

**Fig. 3:**
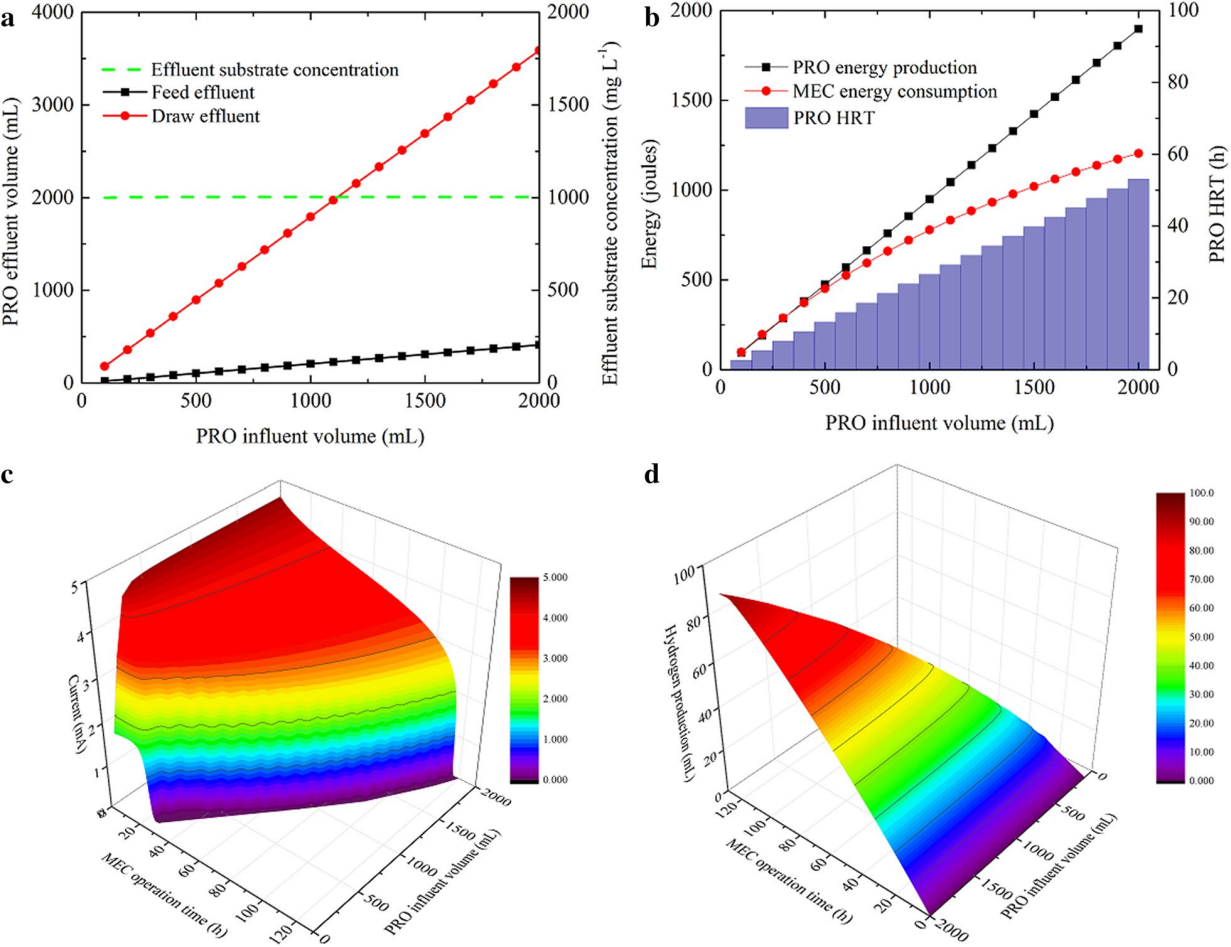
Effects of varied PRO influent volume on **a** PRO effluent volume and substrate concentration, **b** PRO energy production, PRO’s HRT and MEC energy consumption, **c** current generation in the MEC and **d** hydrogen production in the MEC.

In theory, the osmotic pressure difference will be less affected by the water flux at larger volumes of the feed and the draw solutions, leading to a prolonged PRO process and more energy available for driving the MEC. This hypothesis was examined with the PRO influent volume varied from 100 to 2,000 mL (simultaneous change in both the feed and the draw volumes), with other parameters listed in the Table 1 and 0.5 LMH as a simulation end-point. The PRO simulation shows that both water recovery and energy production are enhanced by increasing the PRO influent volume, while the substrate concentration in the feed effluent remains stable (Fig. 3a, b). At the PRO influent of 1,000 mL, 794 mL of clean water is recovered and 949 J of energy is produced. These values are increased by two times with 2,000 mL of PRO influent. As the initial substrate concentration in the PRO feed influent remains unchanged (193 mg L^−1^), increasing the PRO influent volume leads to a higher total organic content in the MEC anolyte, which prolongs the MEC’s HRT but does not exert effects on the maximum current (4.5 mA, Fig. 3c). As a result of the increased organics and HRT, hydrogen production is improved consistently. It is predicted that the PRO-MEC system produces 59 mL of H_2_ in 77.7 h with 1,000 mL of the PRO influent, and 92 mL of H_2_ in 121.2 h with 2,000 mL of the PRO influent (Fig. 3d). Interestingly, the energy consumption by the MEC increases non-linearly with increased PRO influent volume, resulting in an enlarged energy surplus between PRO unit and MEC (Fig. 3b). This is indicative that one PRO unit can drive multiple MECs at large volume loading, thereby further enhancing the overall treating capacity of the PRO-MEC system.

#### Effects of NaCl concentration in the PRO draw influent

**Fig. 4:**
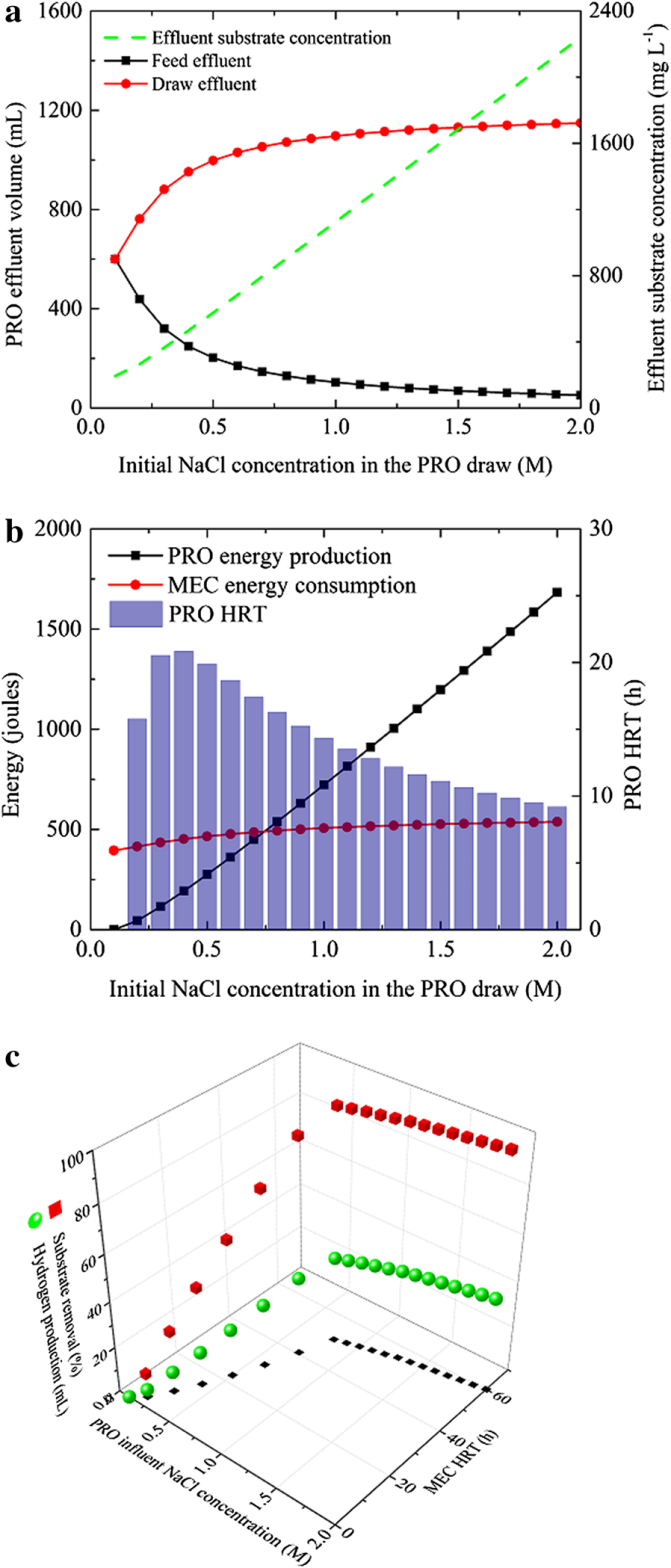
Effects of varied NaCl concentration on **a** PRO effluent volume and substrate concentration, **b** PRO energy production, PRO’s HRT and MEC energy consumption and **c** substrate removal and hydrogen production in the MEC.

The NaCl concentration ranging from 0.1 to 2.0 M is input in the time-dependent PRO model with other parameters summarized in the Table 1 and 0.5 LMH as simulation end-point. It is predicted that the water recovery is enhanced significantly when the NaCl concentration increases from 0.1 to 0.7 M, and further increase in the NaCl concentration does not result in continuing improvement of water recovery (Fig. 4a). On the other hand, both the energy production and the effluent substrate concentration increase linearly with the increased NaCl concentration (Fig. 4a, b). The PRO’s HRT reaches the maximum of 20.8 h at 0.4 M, and then decreases readily to 9.2 h at 2.0 M (Fig. 4b), likely because high water flux at a higher draw concentration causes faster decline in the osmotic pressure difference as described by Eqs. 5 and 6. When the NaCl concentration is lower than 0.7 M, the PRO energy production is predicted to be lower than the MEC energy consumption if the substrate is completely degraded (Fig. 4b). Therefore, the MEC performance is limited by the energy supply with low NaCl concentrations, and both the organics removal and hydrogen production cannot be performed effectively (below 93.0 % and 34.2 mL, respectively, Fig. 4c). When the NaCl concentration is higher than 0.8 M, sufficient electrical energy is generated in the PRO unit to support a complete MEC cycle with organics removal >99 %. Consequently, the MEC’s operating time is prolonged to over 50.7 h, and the hydrogen production reaches 37.6 mL. Further increase in the NaCl concentration beyond 1.5 M does not noticeably enhance the hydrogen production, suggesting that the total organic content may have become a limiting factor for the MEC performance.

#### Effects of external voltage on the MEC

**Fig. 5:**
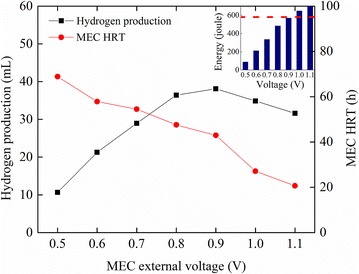
Hydrogen production and MEC’s HRT predicted by the batch-mode MEC model with external voltage ranging from 0.5 to 1.1 V. The *inset* shows the MEC energy consumption at different voltage and the red line indicates the PRO energy production.

The energy consumption by the MEC is affected by the externally applied voltage according to Eqs. 18 and 19. MEC simulation is therefore performed with external voltage varying from 0.5 to 1.1 V. The initial conditions for the MEC simulation are listed in Table 1. The results show that at 0.5 V, the MEC needs 89 J of energy to remove organics, but generates only 10.7 mL H_2_ in 68.8 h (Fig. 5). The hydrogen production reaches the maximum value of 38.1 mL in 42.9 h at 0.9 V, and declines at a higher external voltage. That is because the MEC energy consumption exceeds the PRO energy production: the PRO energy production with given input parameters is fixed (i.e. 579 J, Fig. 5 inset, red dash line), and at 1.0 V (or 1.1 V), the MEC simulation is stopped once the energy consumption reaches 579 J; as a result, the MEC cannot accomplish a complete cycle and hydrogen production would decrease because of a shorter operating time. The organic removal is predicted to be 92.7 % at 1.0 V and only 80.3 % at 1.1 V. Meanwhile, the MEC’s HRT is significantly shortened at a higher external voltage. These results indicate that the coupled system is versatile towards different purposes, and the treating capacity of the PRO and MEC can be balanced through varying external voltage.

### Perspectives

The coupled PRO-MEC system can achieve multiple benefits. Firstly, the PRO unit can serve as pre-treatment of organic solutions (e.g. wastewater), substantially reducing its volume and extracting clean water [15]. The draw solution for the PRO unit could be brine from RO desalination (91.3 % of water recovery predicted by the model) or seawater (71.7 % water recovery). Secondly, the PRO process can generate a high-conductivity feed solution as the MEC anolyte, which decreases the electrolyte resistance and thus is beneficial for bioelectrochemical processes [35, 36]. Thirdly, the osmotic energy harvested by the PRO process at certain influent volume and draw concentration is sufficient for a complete MEC cycle, making organic removal and hydrogen production more sustainable. Fourthly, compared with other approaches (e.g., MFC or solar energy) for driving hydrogen production in MECs, the present system can reduce the volume of the treated wastewater and produce a stream (diluted draw solution) for further desalination. For example, when seawater serves as a draw solution, its conductivity can be decreased from 54.7 to 32.0 mS cm^−1^ after the PRO process. Our previous study suggests that such a dilution can significantly enhance the rate of conductivity reduction in a microbial desalination cell [25].

Despite the great promise, several challenges must be addressed to move the PRO-MEC system towards practice. First, the robustness of the batch-mode MEC model should be improved by introducing endogenous respiration and more accurate pH variables [37, 38]. Because buffer was added in the feed/anolyte, the equilibrium between [HCO_3_^−^] and [H_2_CO_3_] as a function of pH was not taken into account in this study. When real wastewater is used as a feed/anolyte, the equilibrium constant should be introduced in Eqs. 14 and 15. Moreover, the cathode potential in Eq. 16 could be modified by relating pH change to hydrogen production and the Fick’s law of diffusion. Another critical issue for practical applications is to balance the treating capacity of PRO and MEC. The simulation results with a high PRO influent volume and a high draw concentration suggest that one PRO unit is able to generate surplus energy to drive multiple MECs. In addition, the disparity in HRTs between the PRO unit and the MEC should also be addressed with caution to optimize the efficiency when one PRO unit is coupled to several MECs. Finally, the proposed system needs to be further demonstrated with actual energy generated from a PRO unit, which was reported in only a few studies [39, 40].

## Conclusions

This work has demonstrated a proof-of-concept PRO-MEC system for simultaneous bioelectrochemical hydrogen production, organic removal, and water extraction driven by two forms of energy generated from (theoretically) the same liquid stream. It also presents the first attempt to introduce time variable into a PRO model, and has modified the previous single-chamber MEC model into a two-chamber batch-mode MEC model. Multiple benefits can be achieved through the synergy between PRO and MEC, including sustainable hydrogen production, clean water recovery and reduced wastewater volume. A high influent volume and a high draw concentration are predicted to enhance the performance of the coupled system, and the optimal external voltage is determined to be 0.9 V by the models. The PRO-MEC system may hold great promise in addressing water-energy nexus.

## Methods

### System setup and operation

The experimental system consisted of a PRO unit and an MEC, as shown in Fig. 1. A forward osmosis (FO) cell (Sepa CF II Forward Osmosis Cell 316 SS, Sterlitech Corporation, USA) was used as the PRO unit, containing an FO membrane (HTI OsMem™ CTA-ES, Hydration Technology Innovations, USA) tailored to an area of 0.014 m^2^. The feed solution contained (per liter of deionized water): sodium acetate, 193 mg; NaCl, 0.5 g; MgSO_4_, 0.015 g; CaCl_2_, 0.02 g; KH_2_PO_4_, 1.06 g; K_2_HPO_4_, 2.14 g; NaHCO_3_, 1 g; and trace element, 1 mL. A synthetic organic solution was used because it could be better controlled for organic concentrations and other parameters. The feed solution (600 mL, π_F,0_ = 2.4 bar) was recirculated between a reservoir and the PRO feed chamber by a peristaltic pump (Langer Instruments Corp., USA) at a cross-flow velocity of 16.4 cm s^−1^. The NaCl solution (0.8 M, 600 mL, π_D,0_ = 36.2 bar) was used as a draw and recirculated between a reservoir and the PRO draw chamber by a high pressure water pump (Estone 12 V DC 5 L/min 60 W, China) at a cross-flow velocity of 85.9 cm s^−1^. Low hydraulic pressures (1, 2 and 3 bar) were applied on the draw chamber of the PRO cell using a pressure valve at the outlet and monitored with two gauges at both inlet and outlet. The PRO unit was operated for 6.0 h as a batch, and the weight change in both feed and draw solutions was monitored by using digital balances (Scort Pro, Ohaus, USA).

A two-chamber MEC was built as previously described [41], with a carbon-brush anode electrode and a carbon-cloth cathode electrode that contained 5 mg cm^−2^ of Pt/C and a surface area of 10 cm^2^. The anode was inoculated with anaerobic sludge from a local wastewater treatment plant (Peppers Ferry, Radford, VA, USA). The liquid volumes of the anode compartment and the cathode compartment of the MEC were equal at 140 mL/each. Those electrolytes were recirculated between the MEC and the external reservoirs. An external voltage of 0.8 V was applied on the MEC with a power supply (3644A, Circuit Specialists, Inc., USA) to mimic the utilization of the energy from the PRO unit. The electrolytes were prepared as shown in Table 1 and purged with nitrogen gas for 15 min prior to each test. The MEC was operated under a batch mode at room temperature. The operation time of the MEC was determined by equating the PRO energy production with the MEC energy consumption by assuming no energy loss during energy conversion process. In both the PRO and MEC experiments, triplicate experiments were conducted for each test condition. The system at different PRO influent volumes, NaCl concentrations and external voltages was evaluated with the time-dependent PRO model and the batch-mode MEC model.

### Measurement and analysis

Weight change of the feed and draw solutions was recorded by an auto logging software (Scort Pro, Ohous, USA) at a time interval of 2 min. Water flux was calculated as derivative of the volume change. Conductivity was measured by using a benchtop conductivity meter (Mettler-Toledo, USA). The voltage (*U*) of the MEC on a resistor (*R* = 1 Ω, HARS-X-3_0.001, USA) was recorded by a digital multimeter (2700, Keithley Instruments, Inc., USA) at a time interval of 5 min. The current was calculated according to Ohm’s law: *i* = *U*/*R*. Hydrogen produced by the MEC was collected using water displacement method and the volume was measured with a syringe. COD was measured using a DR/890 colorimeter (HACH Co., Ltd., USA) according to manufacturer’s instruction. Organics (acetate) concentration in the effluent was calculated by dividing the COD by the empirical coefficient of 0.78. The pH was measured by a benchtop pH meter (Oakton Instruments, USA). Important MEC parameters were calculated as previously described [41]. Hydrogen production rate was calculated based on the catholyte volume, which was obtained from the PRO simulation.

### Time-dependent PRO model

The time-dependent water flux was simulated based on the PRO model developed by Yip et al. [27]:

1 $$J_{t} = A\left\{ {\frac{{\pi_{D,t} \exp \left( { - \frac{{J_{t} }}{k}} \right) - \pi_{F,t} \exp \left( {\frac{{J_{t} S}}{D}} \right)}}{{1 + \frac{B}{{J_{t} }}\left[ {\exp \left( {\frac{{J_{t} S}}{D}} \right) - \exp \left( { - \frac{{J_{t} }}{k}} \right)} \right]}} - P_{t} } \right\}$$

where *J*_*t*_ (m^3^ m^−2^ s^−1^) is the instantaneous water flux at time *t*, *π*_*D,t*_ and *π*_*F,t*_ (bar) are the osmotic pressure of the bulk draw and feed solution at time *t*, respectively, *A* (m^3^ m^−2^ s^−1^ bar^−1^) is the permeability coefficient, *B* (m^3^ m^−2^ s^−1^) is the salt permeability coefficient, *S* (m) is the support layer structural parameter, *D* (m^2^ s^−1^) is the diffusion coefficient of NaCl in the membrane substrate, *k* (m^3^ m^−2^ s^−1^) is the mass transfer coefficient, and *P*_*t*_ (bar)is the hydraulic pressure applied on the draw chamber at time *t*. The real-time volume of the feed (*V*_*F,t*_) and draw (*V*_*D,t*_) are thus:

2 $$V_{F,t} = V_{F,0} - \int {J_{t} dt}$$

3 $$V_{D,t} = V_{D,0} + \int {J_{t} dt}$$

where *V*_*F,0*_ and *V*_*D,0*_ (L) are the initial feed and draw volume. The reverse salt flux *J*_*S,t*_ (mol m^−2^ s^−1^) can be approximated as [42]:

4 $$J_{S,t} = \frac{{BJ_{t} }}{AiRT}$$

where *i* is the number of dissolved species (2 for NaCl), *R* (8.3145 J mol^−1^ K^−1^) is the ideal gas constant, and *T* (298.15 K) is the absolute temperature. According to the van’t Hoff equation: $\pi = iRTC$, where *C* (M) is the molar concentration, the real-time osmotic pressure is obtained by combining Eqs. 1–4:

5 $$\pi_{F,t} = iRT\left( {\frac{{V_{F,0} C_{F,0} + a\int {J_{S,t} dt} }}{{V_{F,t} }}} \right)$$

6 $$\pi_{D,t} = iRT\left( {\frac{{V_{D,0} C_{D,0} - a\int {J_{S,t} } dt)}}{{V_{D,t} }}} \right)$$

where *a* (0.014 m^2^) is the membrane area, *C*_*F*,*0*_ (M) is the initial salt concentration in the feed solution obtained by multiplying the conductivity (mS cm^−1^) by the empirical coefficient of 0.64 ppm and dividing the result by the molecular weight of NaCl (58.5 g mol^−1^), and *C*_*D*,*0*_ (M) is the initial NaCl concentration. It can be derived from the expression of power density and ideal water flux that the maximum power is generated when *P* = *(π*_*D*_ − *π*_*F*_*)*/2 [16]. In order to maintain the maximum energy production *Q*_*PRO*_ (joule) during the PRO process, the instantaneous hydraulic pressure applied on the draw chamber in Eq. 1 is calculated as:

7 $$P_{t} = \frac{{\left( {\pi_{D,t} - \pi_{F,t} } \right)}}{2}$$

8 $$Q_{PRO} = a\int {P_{t} J_{t} dt}$$

### Batch-mode MEC model

The batch-mode MEC model was modified based on previously reported multi-population MEC model and MDC model [28, 29, 43]. Because acetate was used as substrate and the cathode chamber is abiotic, it was assumed that the anode biofilm was composed of acetoclastic methanogens and exoelectrogens. Moreover, the MEC was operated under a batch mode and thus the dilution rate used in the previous study is not applicable [28]. The mass balance for the substrate and the growth of the organisms can be written:

9 $$\frac{dS}{dt} = - q_{e,max } \frac{S}{{K_{e} + S}}\frac{{M_{OX} }}{{K_{M} + M_{OX} }}x_{e} - q_{m,{max} } \frac{S}{{K_{m} + S}}x_{m}$$

10 $$\frac{{dx_{e} }}{dt} = - \mu_{e, {max} } \frac{S}{{K_{e} + S}}\frac{{M_{OX} }}{{K_{M} + M_{OX} }}x_{e} - d_{e} x_{e}$$

11 $$\frac{{dx_{m} }}{dt} = - \mu_{m,max} \frac{S}{{K_{m} + S}}x_{m} - d_{m} x_{m}$$

12 $$\frac{{dM_{OX} }}{dt} = - Y_{M} \frac{S}{{K_{e} + S}}\frac{{M_{OX} }}{{K_{M} + M_{OX} }} + \frac{\gamma }{{V_{a} x_{e} }}\frac{I}{nF}$$

where *S* (mg-S L^−1^) is the acetate concentration, *x*_*e*_ and *x*_*m*_ (mg-x L^−1^) are the concentrations of exoelectrogens and methanogens, respectively, *q*_*e,max*_ and *q*_*m,max*_ (mg-S mg-x^−1^ day^−1^) are the maximum substrate consumption rates by organisms, *K*_*e*_, *K*_*m*_, and *K*_*M*_ (mg-S L^−1^, mg-S L^−1^, and mg-M mg-x^−1^) are the half saturation concentrations for exoelectrogens, methanogens, and the redox mediators, respectively, *µ*_*e,max*_ and *µ*_*m,max*_ (day^−1^) are the maximum growth rates by the organisms, *d*_*e*_ and *d*_*m*_ (day^−1^) are the decay rates of the organisms, *M*_*OX*_ (mg-M mg-x^−1^) is the fraction of oxidized mediators per exoelectrogen, *Y*_*M*_ (mg-M mg-S^−1^) is the mediator yield, *γ* (mg-M mole-M^−1^) is the mediator molar mass, *I* (A) is the current through the circuit of the MEC, *F* (96,485 C mol^−1^) is the Faraday constant, *n* (2) is number of electrons transferred per mole of mediator, *V*_*a*_ (L) is the anolyte volume. The anode and cathode potential *E*_*A*_ and *E*_*C*_ (V) are calculated with the Nernst equation [44]:

13 $$E_{A} = E_{A}^{0} - \frac{RT}{8F}In\frac{S}{{\left[ {HCO_{3}^{ - } } \right]^{2} \left[ {H^{ + } } \right]^{9} }}$$

14 $$\left[ {HCO^{ - }_{3} } \right] = \left[ {HCO^{ - }_{3} } \right]_{0} \frac{{V_{F,0} }}{{V_{F} }} + \frac{{2\left( {S_{0} - S} \right)}}{{m_{S} }}$$

15 $$\frac{{d\left[ {H^{ + } } \right]}}{dt} = \frac{\beta }{{m_{S} }}\frac{dS}{dt}\left( {9 - 8 \cdot CE \cdot Y_{HZ} } \right)$$

16 $$E_{C} = E_{C}^{0} - \frac{RT}{2F}In\frac{1}{{\left( {10^{ - 11} } \right)^{2} }}$$

where $E_{A}^{0}$ (0.187 V) is the standard reduction potential of HCO_3_^−^/CH_3_COOH^−^ and $E_{C}^{0}$ (0 V) is the standard reduction potential of H^+^/H_2_, [HCO_3_^−^]_0_ (0.00012 M) is the initial bicarbonate concentration in the feed influent, *V*_*F,0*_ and *V*_*F*_ (L) are the initial and final volume of the PRO feed, *S*_*0*_ and *S* (mg L^−1^) are the initial and final acetate concentration in the PRO feed, *m*_*S*_ (82 g mol^−1^) is the molar weight of acetate, *β* (%) is the buffer efficiency of the anolyte, *CE* (%) is the coulombic efficiency and *Y*_*H2*_ (%) is the cathodic efficiency. The rationale of Eq. 15 is that every mole of the acetate produces 9 mol of protons and 8 mol of electrons. With 8 *CE* moles of electrons being transferred to the cathode, *8 CE Y*_*H2*_ mol of protons migrate and diffuse to the cathode and are reduced to H_2_, leaving the residual protons to affect the anolyte pH. The cathode potential is assumed to be stable and calculated with a pH value of 11 (10^−11^ in Eq. 16 as proton concentration) because no buffer is added in the draw solution/catholyte, and the pH increased from neutral to 11.64 ± 0.02 in the first 3 h and remained stable throughout the MEC experiments. At batch mode, the concentration overpotential *η*_*con*_ (V) in the anode is determined by both the mediator concentration and the substrate concentration, and thus can be written:

17 $$\eta_{con} = \frac{RT}{F}In\frac{{M_{Total} }}{{M_{Total} - M_{OX} }}\frac{{S_{0} }}{S}$$

where *M*_*Total*_ (mg-M mg-x^−1^) is the total mediator fraction per exoelectrogen. Combining Eqs. 13–17 yields the MEC current, the energy consumption *Q*_*MEC*_ (joule) and the hydrogen production *V*_*H2*_ (mL):

18 $$I = \frac{{\left( {E_{C} - E_{A} } \right) + E_{ext} - \eta_{con} }}{{\text{R}_{ext} + R_{in} }}\frac{{M_{Total} - M_{OX} }}{{\varepsilon + M_{Total} - M_{OX} }}$$

19 $$Q_{MEC} = E_{ext} \int {Idt}$$

20 $$V_{H2} = Y_{H2} \frac{I}{2F}\frac{RT}{P}$$

where *E*_*ext*_(V) is the external voltage applied on the MEC, *R*_*ext*_(Ω) is the external resistance, ε (0.0001 mg-M mg-x^−1^) is a constant from a previous study [43], *P* (1 atm) is the air pressure in the cathode and *R*_*in*_ (Ω) is the internal resistance [43]:

21 $$R_{in} = R_{min} + (R_{max} - R_{min} )e^{{ - K_{R} x_{e} }}$$

where *R*_*min*_ (Ω) is the lowest observed internal resistance, *R*_*max*_ (Ω) is the highest observed internal resistance, and *K*_*R*_ (L mg-x^−1^) is the constant that determines the curve steepness.

### Parameter estimation

The parameters were estimated as previously described and listed in the Additional file 2 [28]. The relative root-mean square error (RMSE) as a measure of the discrepancy between the experimental data and the simulation results was calculated:

22 $$RMSE = \frac{{\sqrt {\frac{{\sum\nolimits_{i = 1}^{N} {(y_{i} - \hat{y}_{i} )^{2} } }}{N}} }}{{\hat{y}_{i,max} }}$$

where *N* is the total sampling time points in the simulation; *ŷ*_*i*_ and *y*_*i*_ are experimental data and model predicted values at *t*, respectively; and *ŷ*_*i*,*max*_ is the maximum value of the experimental data. The RMSE of the PRO model was calculated using the feed volume, and that of the MEC model was calculated using electricity.

## Additional files

Additional file 1: Experimental data and simulation results of draw (increase over time), feed volume (decrease over time) and water flux at (A) 1 bar, (B) 2 bar and (C) 3 bar. Additional file 2: Parameters used in the time-dependent PRO model and batch-mode MEC model. Additional file 3: Experimental data and simulation results of draw (increase over time), feed volume (decrease over time) and water flux with (A) 0.5 and (B) 2.0 M NaCl. Additional file 4: (A) Experimental data and simulation result of current generation in the MEC with 357 and 2007 mg L^−1^ substrate in the MEC anolyte, and at the external voltage of (B) 0.6 and (C) 1.0 V.

## Abbreviations

FO: forward osmosis
PRO: pressure-retarded osmosis
BES: bioelectrochemical systems
MEC: microbial electrolysis cell
MFC: microbial fuel cell
OsMFC: osmotic microbial fuel cell
MDC: microbial desalination cells
HRT: hydraulic retention time
LMH: L m^−2^ h^−1^
RMSE: root-mean square error
