# Supplementary figures and images for: Bioelectrochemical production of hydrogen in an innovative pressure-retarded osmosis/microbial electrolysis cell system: experiments and modeling
Source: Biotechnol Biofuels. 2015 Aug 14;8:116. doi: 10.1186/s13068-015-0305-0 (PMC4535853; doi:10.1186/s13068-015-0305-0)

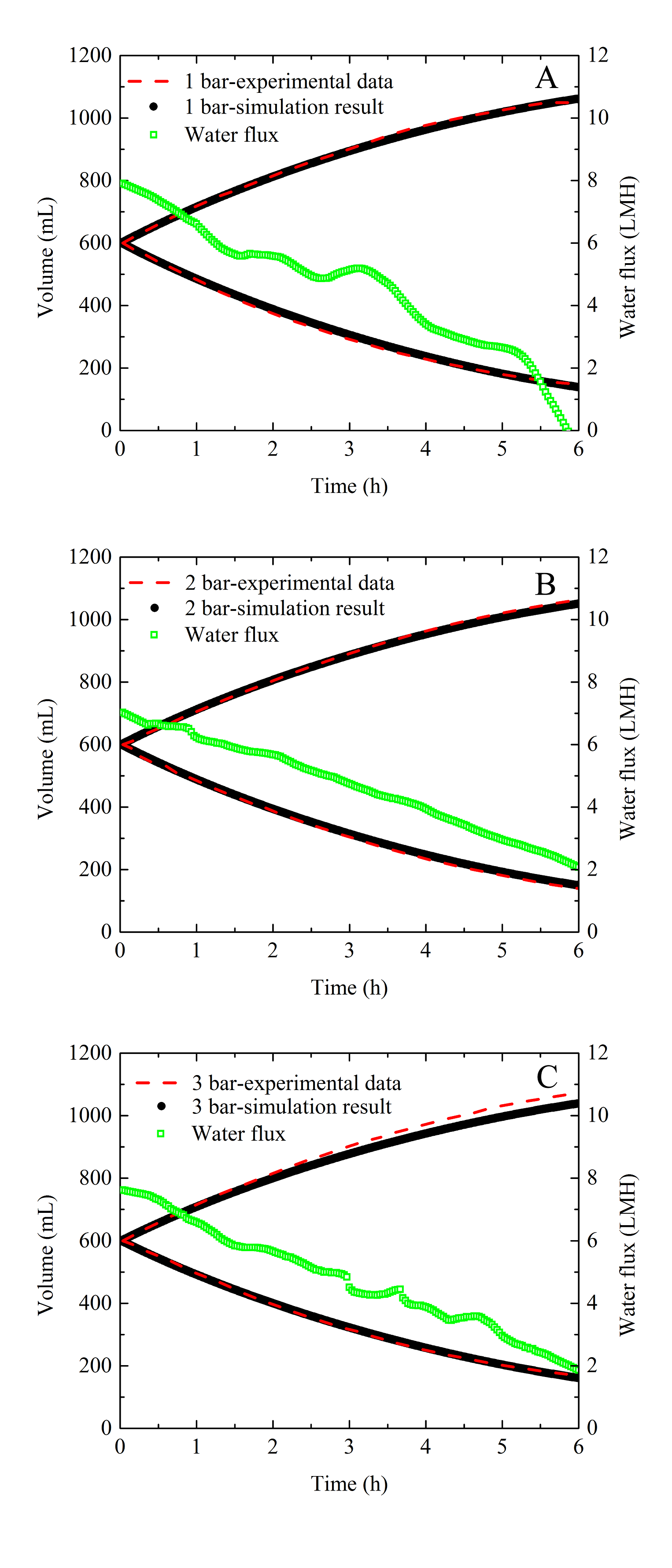

Supplement: Additional file 1: — Experimental data and simulation results of draw (increase over time), feed volume (decrease over time) and water flux at (A) 1 bar, (B) 2 bar and (C) 3 bar. [file 13068_2015_305_MOESM1_ESM.tif]

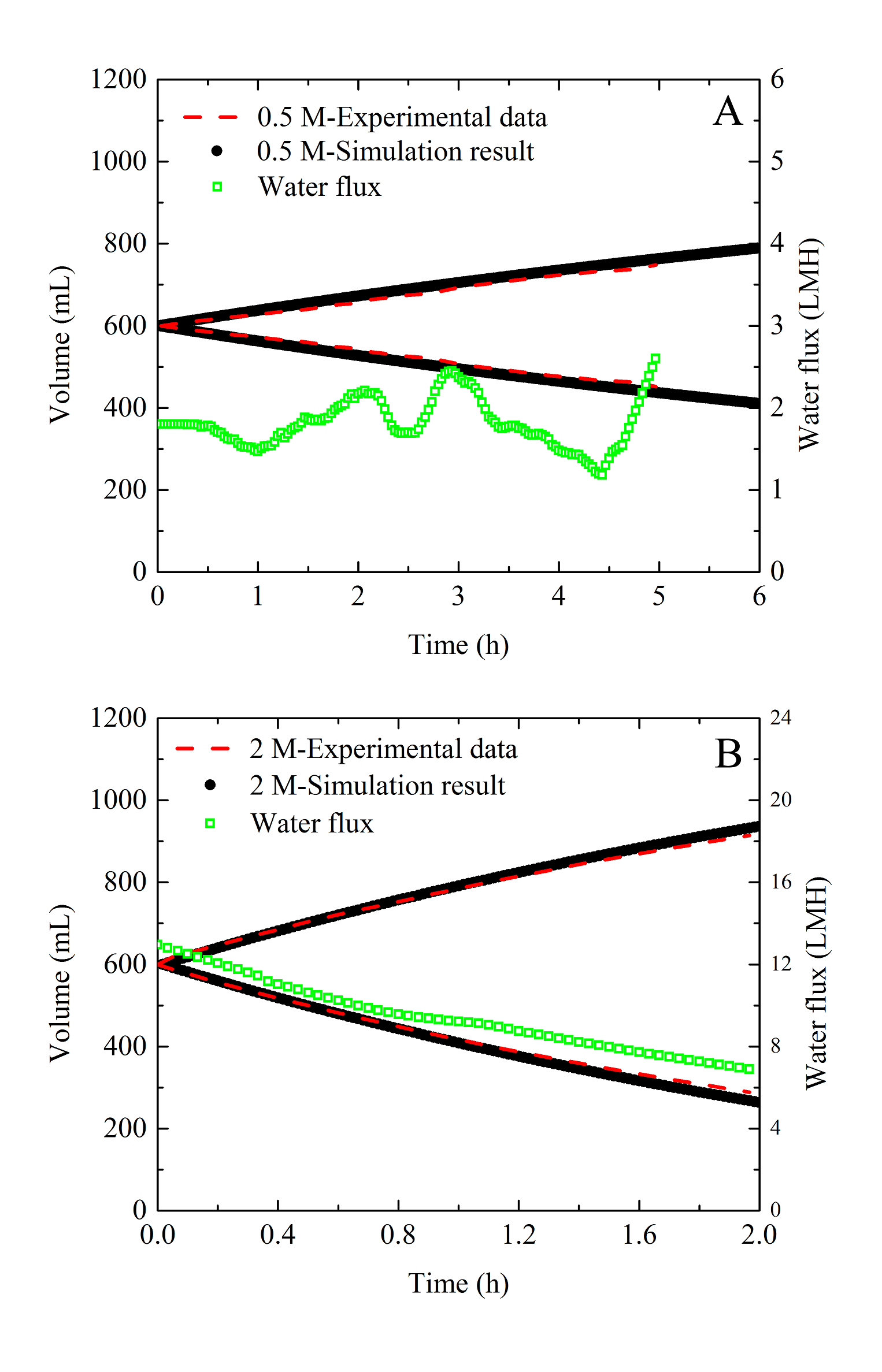

Supplement: Additional file 3: — Experimental data and simulation results of draw (increase over time), feed volume (decrease over time) and water flux with (A) 0.5 and (B) 2.0 M NaCl. [file 13068_2015_305_MOESM3_ESM.tif]

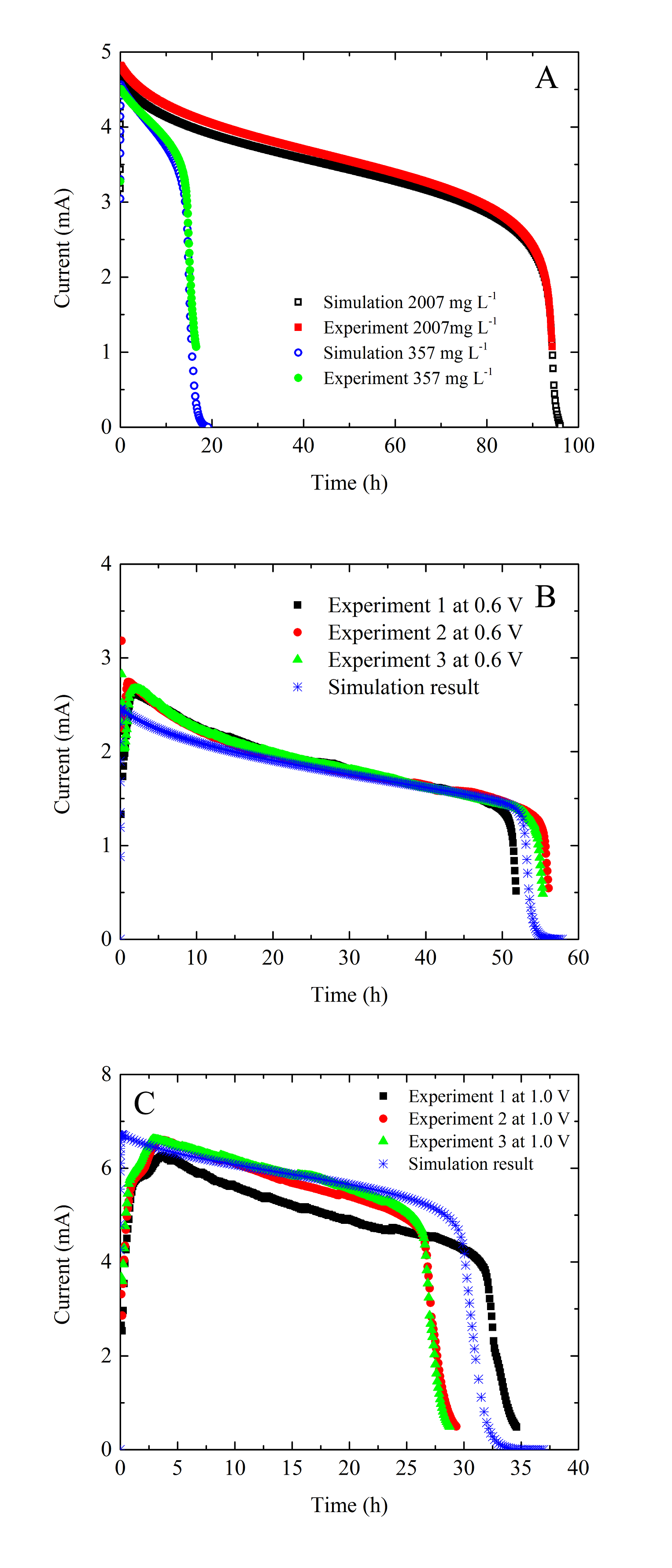

Supplement: Additional file 4: — (A) Experimental data and simulation result of current generation in the MEC with 357 and 2007 mg L−1 substrate in the MEC anolyte, and at the external voltage of (B) 0.6 and (C) 1.0 V. [file 13068_2015_305_MOESM4_ESM.tif]
