# Supplementary material for: Bioelectrochemical production of hydrogen in an innovative pressure-retarded osmosis/microbial electrolysis cell system: experiments and modeling
Source: Biotechnol Biofuels. 2015 Aug 14;8:116. doi: 10.1186/s13068-015-0305-0 (PMC4535853; doi:10.1186/s13068-015-0305-0)
Supplement: Additional file 2: — Parameters used in the time-dependent PRO model and batch-mode MEC model. [file 13068_2015_305_MOESM2_ESM.docx]

**Parameters used in the time-dependent PRO model and the batch-mode MEC model.**

| **Parameters** | | **Description** | **Value** |
| --- | --- | --- | --- |
| **PRO** | *A* | permeability coefficient | 1.20×10^-7^ m^3^ m^-2^ s^-1^ bar^-1^ |
|  | *B* | salt permeability coefficient | 1.39×10^-7^ m^3^ m^-2^ s^-1^ |
|  | *S* | support layer structural parameter | 0.0005 m |
|  | *D* | diffusion coefficient of NaCl in the membrane substrate | 1.48×10^-9^ m^2^ s^-1^ |
|  | *k* | mass transfer coefficient | 1.48×10^-5^ m^3^ m^-2^ s^-1^ |
|  | *i* | number of dissolved species | 2 |
|  | *R* | ideal gas constant | 8.3145 J mol^-1^ K^-1^ |
|  | *a* | membrane area | 0.014 m^2^ |
| **MEC** | *x_e,max_* | maximum concentrations of exoelectrogens | 438.9 mg-x L^-1^ |
|  | *x_m,max_* | maximum concentrations of methanogens | 10 mg-x L^-1^ |
|  | *q_e,max_* | maximum substrate consumption rates by exoelectrogens | 3 mg-S mg-x^-1^ day^-1^ |
|  | *q_m,max_* | maximum substrate consumption rates by methanogens | 10 mg-S mg-x^-1^ day^-1^ |
|  | *K_e_* | half saturation concentrations for exoelectrogens | 35 mg-S L^-1^ |
|  | *K_m_* | half saturation concentrations for methanogens | 80 mg-S L^-1^ |
|  | *K_M_* | half saturation concentrations for redox mediators | 0.01 mg-M mg-x^-1^ |
|  | *µ_e,max_* | maximum growth rates by the exoelectrogens | 0.197 day^-1^ |
|  | *µ_m,max_* | maximum growth rates by the methanogens | 0.1 day^-1^ |
|  | *M_total_* | total mediator fraction per exoelectrogen | 0.05 mg-M mg-x^-1^ |
|  | *Y_M_* | mediator yield | 22 mg-M mg-S^-1^ |
|  | *γ* | mediator molar mass | 663400 mg-M mole-M^-1^ |
|  | *F* | Faraday constant | 96485 C mol^-1^ |
|  | *β* | buffer efficiency of the anolyte | 9.2730711×10^-7^ |
|  | *CE* | coulombic efficiency | 0.5731 |
|  | *Y_H2_* | cathodic efficiency | 0.4748 |
|  | *R_min_* | the lowest observed internal resistance | 90 Ω |
|  | *R_max_* | the highest observed internal resistance | 2000 Ω |
|  | *K_R_* | a constant that determines the curve steepness | 0.0818 L mg-x^-1^ |
|  | *ε* | a constant that affects the current | 0.0001 mg-M mg-x^-1^ |
